# The effect of meteorological factors on adolescent hand, foot, and mouth disease and associated effect modifiers

**DOI:** 10.3402/gha.v7.24664

**Published:** 2014-08-05

**Authors:** Haixia Wu, Hongchun Wang, Qingzhou Wang, Qinghua Xin, Hualiang Lin

**Affiliations:** 1 State Key Laboratory for Infectious Diseases Prevention and Control, National Institute for Communicable Disease Control and Prevention, Chinese Center for Disease Control and Prevention, Beijing, China; 2 Qilu Hospital, Shandong University, Jinan, China; 3 Shandong Academy of Occupational Health and Occupational Medicine, Jinan, China; 4 Guangdong Provincial Institute of Public Health, Guangdong Provincial Center for Disease Control and Prevention, Guangzhou, China

**Keywords:** hand, foot and mouth disease, weather, mean temperature, relative humidity

## Abstract

**Background:**

Hand, foot, and mouth disease (HFMD) is a contagious viral illness that commonly affects infants and children. This infection is an emerging infectious disease in Rizhao in recent years. The present study examined the short-term effects of meteorological factors on adolescent HFMD in Rizhao.

**Design:**

A generalized additive Poisson model was applied to estimate the effects of meteorological factors on adolescent HFMD occurrence in 2010–2012. Subgroup analyses were also conducted to examine the potential effect modifiers of the association in terms of age, sex, and occupation.

**Results:**

A positive effect of temperature was observed (ER [excess risk]=1.93%, 95% CI: 1.05 to 2.82% for 1°C increase on lag 5 day). A negative effect of relative humidity at lag 1 day and positive effects were found on lag 5–7 days, and an adverse effect was observed for sunshine at lag days 3–4 (ER=−0.71%, 95% CI: −1.25 to −0.17% on lag day 4). We also found that age, sex, and occupation might be important effect modifiers of the effects of weather variables on HFMD.

**Conclusions:**

This study suggests that meteorological factors might be an important predictor of adolescent HFMD occurrence in Rizhao. Age, sex, and occupation might be important effect modifiers of the effects.

Hand, foot, and mouth disease (HFMD) is a viral infectious disease commonly caused by coxsackievirus A16 (Cox A16) and enterovirus 71 (EV71). It is most common among infants and children aged younger than 5 years (1). The main symptom includes fever, painful sores in the mouth, and a rash with blisters on hands, feet, and also buttocks (2). HFMD is primarily transmitted via fecal–oral route and respiratory droplets, contact with blister fluid of infected individuals, or close contact with infected individuals (3). There is no specific drug or vaccine available for HFMD so far, so preventive measures remain the only effective way to prevent its transmission, such as avoiding direct contacts with infective patients, disinfection of contaminated environment, and good personal hygiene habits (4, 5).

Asian countries have experienced an increasing trend of HFMD outbreaks in the past decades, resulting in thousands of deaths among children due to severe complications (4). Particular public health concerns have been raised especially since the severe outbreaks in Malaysia and Taiwan in 1997 and 1998, respectively (4, 6). Several outbreaks have also been witnessed in mainland China in recent years, for example, a total of 1,619,706 new HFMD cases were reported in 2011, resulting in 509 deaths (7).

The incidence of HFMD has presented seasonality in a number of countries. For example, epidemic peaks have been observed during summers in Taiwan and Japan (8, 9). A bimodal seasonal pattern was reported in the United Kingdom with peaks in summer and late autumn/early winter (10). In Finland, most HFMD cases were observed in autumn (11). The seasonality of HFMD incidence indicates that meteorological factors might play an important role on its epidemiology, which has been, however, investigated in a limited number of studies with inconsistent findings. For example, it has been suggested that the changing epidemiology (a new peak in winter) in Hong Kong might be due to increase in winter temperature (12). In Japan, a study found that ambient temperature and relative humidity were associated with increased HFMD occurrence (9). The study in Singapore showed that weekly maximum temperature above 32°C was linked with increased HFMD incidence (4). Time series analysis in Guangzhou and Shenzhen, in China, also found a positive association between temperature and HFMD occurrence (13, 14), whereas, studies in Japan found that the number of days per week with average temperature above 25°C was negatively associated with HFMD incidence (15).

Serious HFMD outbreak has been reported in Rizhao, China, in recent years with peaks observed in summer (16). The current study aimed to examine the relationship of meteorological factors with the occurrence of HFMD in children using surveillance data collected in Rizhao from 2010 to 2012. We also examined whether age, sex, and occupation (scattered children or children at schools) could modify the association between weather factors and the adolescent HFMD risk.

## Methods

### Setting

**Fig. 1:**
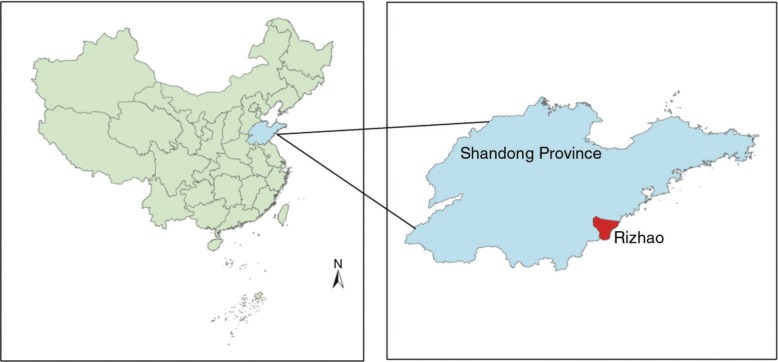
Geographical location of Rizhao in China (left picture shows the location of Shandong Province in China, right shows the location of Rizhao in Shandong Province).

Rizhao is a prefecture-level city in southeastern Shandong Province. According to the 2010 census, its population was 2,801,100 (17). It has a temperate, monsoon-influenced climate. Winter is cool to cold and windy, with a January average temperature of −0.3°C. Summer is generally hot and humid, with an August average temperature of 25.7°C (Fig. 1).

### Data sources

Daily counts of HFMD covering the period 2010–2012 were obtained from Rizhao Municipal Center for Disease Control and Prevention. The diagnosis criteria for HFMD cases were provided in a guidebook published by the Chinese Ministry of Health (18). In brief, patients were diagnosed as HFMD if they had the following symptoms: fever, papules and herpetic lesions on the hands or feet, rashes on the buttocks or knees, inflammatory flushing around the rashes and little fluid in the blisters, or sparse herpetic lesions on oral mucosa. According to China's notifiable infectious disease regulation, all HFMD cases have to be reported to the infectious disease surveillance system. A recent data quality survey demonstrated that the data were of high quality, especially in the eastern regions of China, with reporting completeness of 99.84% and accuracy of the information reported of 92.76% (19). During the study period, more than 99% of the HFMD cases were among the children younger than 18 years old, so we limited our analysis to the HFMD among children aged 0–18 years.

Meteorological data, including daily mean temperature, relative humidity, wind velocity, and rainfall, were obtained from National Weather Data Sharing System, which was publicly accessible.

### Statistical analysis

Spearman's correlation was used to evaluate the inter-correlation between the various weather factors. As daily count of HFMD generally followed a Poisson distribution, a generalized additive model (GAM) with a log link and allowing Poisson auto-regression and over-dispersion was applied to investigate the short-term effects of daily meteorological factors on HFMD (20). We controlled for the day of week (DOW) and public holidays using categorical indicator variables. In addition, we used penalized smoothing splines (21) to adjust for long-term and seasonal trends in daily morbidity with degree of freedom (df) selected a priori based on previous studies (22, 23). For the smooth function of calendar time, 6 df per year was chosen so that we could filter the information at time scales of 2 months (24). The model for temperature can be specified as:

$$\text{log[E(Yt)]}=\alpha +temperature+s(t,df=6/year)+\beta_{\text{1}}*\text{DOW}+\beta_{2}*\mathrm{PH}$$

where E(Yt) is the expected number of HFMD on day t, α is the intercept, s() indicates a smoother based on penalized smoothing splines, df is the degree of freedom, DOW is an indicator for day of week, PH presents a binary variable for the public holiday, and β is the regression coefficient.

We estimated the linear effect of various weather variables according to different lag structures, including current day (lag0) up to 10 days before (lag10) as this infection usually has an incubation period of 3–7 days (25). Univariate model was first fitted for each meteorological factor and then multivariate model was used to control the influence of other meteorological factors. The robustness of the key findings was assessed by changing the df in the smooth function of time used to adjust for seasonal and long-term trends. To justify the assumption of linearity between the logarithm of HFMD count and the key weather variables (with significant association between each other), we visually examined the exposure–response curves derived using a smoothing function (26, 27). We also plotted the residuals (the difference between fitted and observed values) of the model against the time to examine the residual normality and constant variance assumptions.

We tested for effect modification of the significant association by age, sex, and occupation with stratified analyses. We stratified the analyses by age group (≤2 years, 2–18 years), sex (male and female), and occupation (scattered children and schooling children). We classified the children into two groups by age using 2 years old as cut-off point, as children younger than 2 years old could not walk independently (28). By occupation, the children were grouped into scattered children and schooling children, where the scattered children were the pre-school children who have not been to kindergarten or school (29). We tested the statistical significance of differences between the effect estimates of the strata divided by potential effect modifiers by calculating the 95% confidence interval as:

$$(Q_{1}-Q_{2})\pm 1.96\sqrt{{\left(SE_{1}\right)}^{2}+{\left(SE_{2}\right)}^{2}}$$

where Q_1_ and Q_2_ were the effect estimates for each stratum (such as male and female), and SE_1_ and SE_2_ were their corresponding standard errors (30).

All analyses were carried out using the ‘mgcv’ package in the software R (R Development Core Team, 2012) (31). We reported the risk estimation as excess risk (ER), defined as the percentage increase in daily HFMD count for one unit increase in each weather factor.

## Results

During the study period, there were 19,967 HFMD cases among children younger than 18 in the study area. The descriptive summary for daily HFMD and weather conditions has been shown in Table 1. On average, there were 18.3 daily HFMD cases. There were more male cases with a male-to-female sex ratio of 1.62:1 (12,358:7,609); most of the cases were reported among the scattered children. Daily mean temperature, relative humidity, rainfall, wind velocity, and sunshine duration were 13.5°C, 68.4%, 2.1 mm, 1.8 m/s, and 5.9 hours, respectively.

**Table 1:** Summary statistics of daily children HFMD occurrence and weather variables in Rizhao (2010–2012)

Variable	Mean±SD	Min.	P(25)	Median	P(75)	Max.
Daily HFMD cases	18.3±24.6	0.0	2.0	7.0	28.0	170.0
Sex						
Male	11.3±16.1	0.0	1.0	4.0	17.0	99.0
Female	6.9±10.3	0.0	0.0	2.0	10.0	71.0
Age						
0–2 years	8.0±11.7	0.0	0.0	2.0	12.0	72.0
2–18 years	10.2±13.9	0.0	1.0	4.0	14.0	105.0
Occupation						
Scattered	13.1±18.7	0.0	1.0	4.0	20.0	112.0
Schooling	5.2±8.2	0.0	0.0	2.0	6.0	65.0
Temperature (°C)	13.5±9.8	−7.8	4.5	15.1	22.1	31.9
Relative humidity (%)	68.4±20.1	15.0	52.0	71.0	85.0	100.0
Rainfall (mm)	2.1±8.8	0.0	0.0	0.0	0.9	130.2
Wind velocity (m/s)	1.8±0.9	0.0	1.4	1.8	2.4	7.4
Sunshine (hour)	5.9±3.9	0.0	1.8	7.2	9.1	12.8

**Fig. 2:**
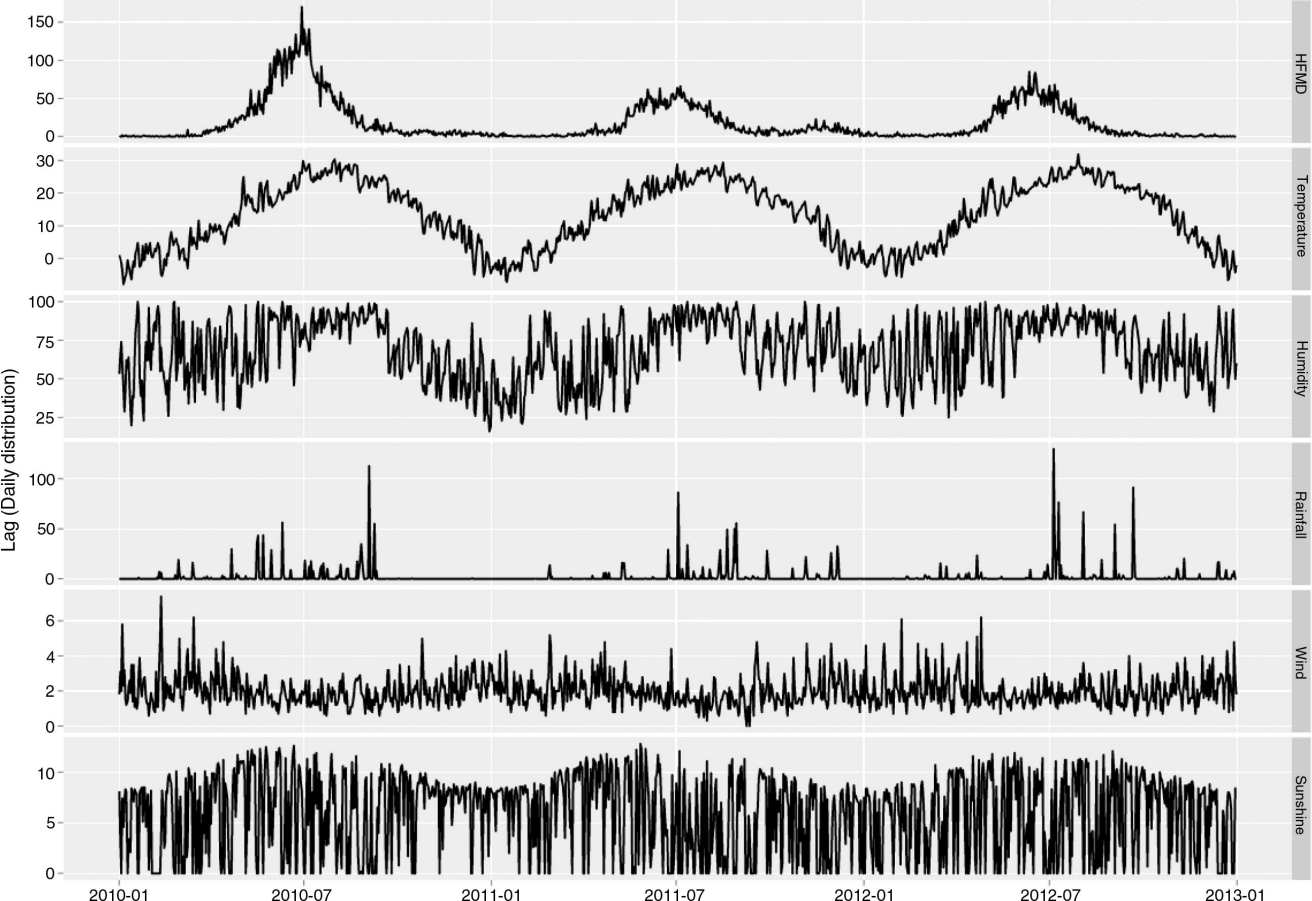
The time series of daily HFMD and weather factors in Rizhao, 2010–2012.

Figure 2 shows the time series of daily HFMD cases and meteorological variables in Rizhao during the study period. There were seasonal patterns in the HFMD occurrence and meteorological factors. Summer epidemic peaks were observed with the most cases in June–August. Table 2 depicts the correlations between various weather variables. All the weather variables were significantly correlated with each other with low to moderate correlation coefficients, for example, between daily mean temperature and relative humidity (*r*=0.54), between relative humidity and rainfall (*r*=0.45).

**Table 2:** Spearman's correlations between daily weather variables in Rizhao, 2010–2012

	Temperature	Humidity	Rainfall	Wind velocity	Sunshine
Temperature	1.00				
Humidity	0.54	1.00			
Rainfall	0.19	0.45	1.00		
Wind velocity	−0.25	−0.33	−0.06	1.00	
Sunshine	0.07	−0.47	−0.47	0.09	1.00

*P*<0.05 for all.

**Fig. 3:**
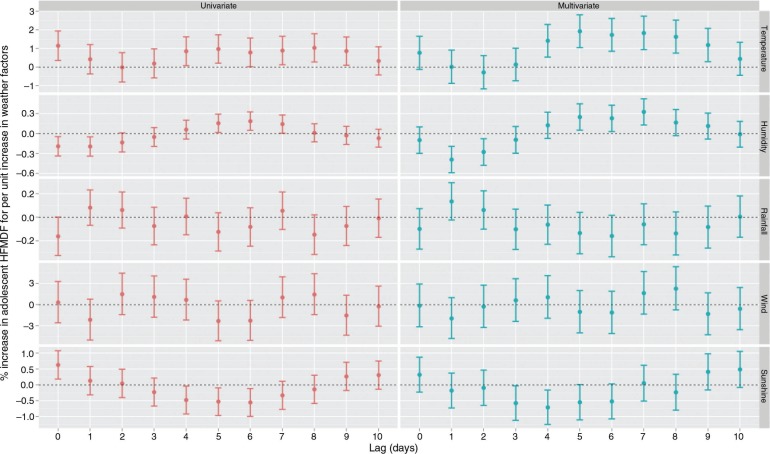
The univariate and multivariate analyses for the association between meteorological variables and adolescent HFMD in Rizhao, 2010–2012 (the effect estimates were excess risk for one unit increase in the weather variables).

The associations between various meteorological factors and adolescent HFMD have been presented in Fig. 3. In the univariate analyses, significant associations were observed for daily mean temperature, relative humidity, and duration of sunshine. Consistent findings were observed in the multivariate model and daily mean temperature at lag of 4–9 days was significantly associated with increased HFMD, with the largest effect observed on lag day 5 (ER=1.93%, 95% CI: 1.05 to 2.82% for one degree increase in daily mean temperature); a negative effect was found for relative humidity on lag days 1–2, with the ER on lag day 1 being −0.39% (95% CI: −0.59 to −0.19% for 1% increase in relative humidity); a positive effect was found for relative humidity on lag days 5–7, with the ER on lag day 7 being 0.33% (95% CI: 0.13 to 0.52%); an adverse effect was observed for sunshine at lag days of 3–4, with the largest effect on lag day 4 (ER=−0.71%, 95% CI: −1.25 to −0.17%). No significant association was observed for rainfall and wind velocity.

Table 3 illustrates the results of stratified analyses by sex, age, and occupation. We found statistically significant difference in the effect of the weather factors. For example, females, children aged 2 years or younger, and scattered children were more sensitive to the effect of temperature; for the effect of relative humidity on lag 1 day, it showed a larger protective effect for females; males, younger children, and schooling children were more vulnerable to the harmful effects of humidity on lag 7 day; and for the effect of sunshine, males and older children benefited more than others.

**Table 3:** Sex, age and occupation-specific excess risk (ER, with 95% CI) of temperature, relative humidity, and duration of sunshine associated children HFMD

	ER (95% CI)			
	
Variable	Temperature (lag 6)	Humidity (lag 1)	Humidity (lag 7)	Sunshine (lag 4)
Sex				
Male	**1.46 (0.44, 2.49)**	−**0.29 (**−**0.52**, −**0.06)**	**0.41 (0.18, 0.64)**	−**0.99 (**−**1.61**, −**0.35)**
Female	**2.11 (0.89, 3.34)**	−**0.53 (**−**0.81**, −**0.25)**	**0.20 (**−**0.07, 0.47)**	−**0.23 (**−**1.00, 0.54)**
Age (years)				
0–2	**2.04 (0.85, 3.24)**	−0.42 (−0.69, −0.16)	**0.50 (0.22, 0.77)**	−**0.02 (**−**0.73, 0.70)**
2–18	**1.32 (0.23, 2.42)**	−0.34 (−0.59, −0.09)	**0.37 (0.12, 0.62)**	−**1.23 (**−**1.92**, −**0.53)**
Occupation				
Scattered	**2.41 (1.41, 3.42)**	−0.42 (−0.65, −0.20)	**0.30 (0.08, 0.53)**	−0.79 (−1.39, −0.19)
Schooling	**1.51 (0.11, 2.93)**	−0.36 (−0.69, −0.03)	**0.45 (0.l3, 0.77)**	−0.94 (−1.89, 0.02)

The bold means statistically significant (*p*<0.05).

Supplementary Fig. 1 shows the diagnostic graph of the multivariate model, illustrating the residuals against time. There were no discernible patterns and no autocorrelation in the residuals, indicating acceptable goodness of fit of the model.

**Fig. 4:**
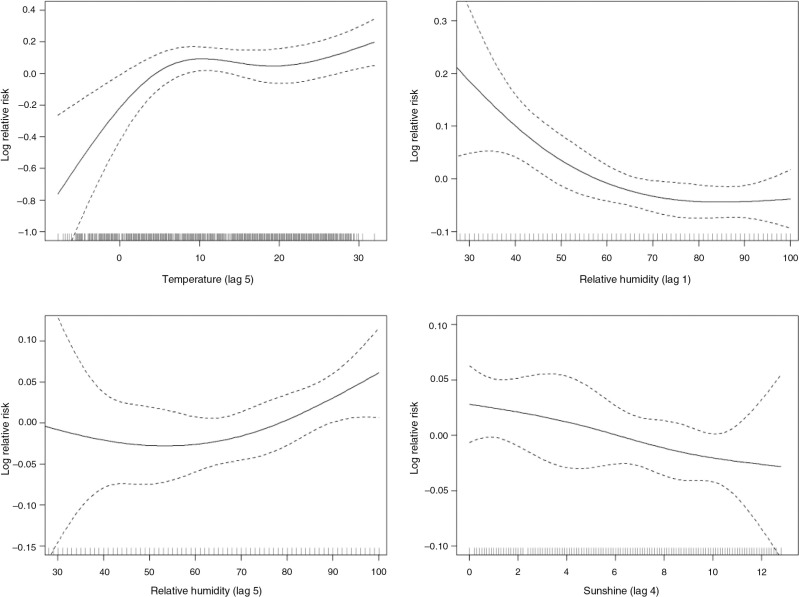
Smoothing plots of daily weather variables against adolescent HFMD in Rizhao, 2010–2012.

The dose–response relationship for temperature, relative humidity, and sunshine with adolescent HFMD occurrence has been presented in Fig. 4. An almost linear relationship was observed for all the variables. Visual inspection of the temperature–HFMD curve suggested that the relative risk increased sharply below 10°C and became relatively flat at 10–20°C with a moderate increase trend afterwards. In the sensitivity analyses, we changed df (4, 5, 7, 8) for calendar time to control for seasonality and long-term trend, which gave similar results.

## Discussion

Time series analysis has been widely used to study the effect of weather variability on human health, particularly some climate-sensitive infectious diseases (32, 33). In the current study, we used a GAM based on Poisson distribution to examine the effect of weather variability on HFMD in the temperate city of Rizhao for the period of 2010–2012. Our results showed that daily mean temperature, relative humidity, and duration of sunshine were significantly related to adolescent HFMD in Rizhao and suggested that age, sex, and occupation might be effect modifiers of the observed association.

The findings of this study were generally consistent with findings from different regions of the world (9, 34, 35). Several postulations could be helpful to understand the possible pathways by which meteorological factors can affect the HFMD occurrence (35, 36). It was possible that ambient temperature, relative humidity, and duration of sunshine could affect the survival and transmission of the HFMD virus in the environment, as well as the behavior and activities of the population, thereby influencing the dynamics of the infection transmission (14).

The present study showed a positive effect of temperature on HFMD incidence, with an increase trend below 10°C and above 20°C and flattening at 10–20°C. Similar finding has also been reported in Tokyo, Japan (9), and Guangzhou, China (13). Contrary to our study, the Japanese study found the breaking temperature point at 20°C, and the study in Guangzhou observed a cutting temperature point at 25°C. This discrepancy might be partially due to the differing temperature profile in these cities.

For relative humidity, we found that it was conversely related with HFMD during the lag 1–2 days, and it was positively associated with increased HFMD risk during longer lay days. Due to the 3–7 days of incubation period of HFMD and in light of previous findings (13), we believed that the positive effect was more biologically plausible, the clinical symptoms were more likely to present several days after the exposure to higher relative humidity, which could facilitate the attachment of the virus on the object surface or toys (25). This feature of HFMD infection also highlighted the importance for more precise modeling in future studies. For instance, compared with the analysis on weekly data, the analysis on daily data is more likely to capture more precise association between weather variables and the disease occurrence.

Consistent with previous studies in Tokyo and Guangdong (15, 37), the current study found an adverse effect of daily sunshine duration in Rizhao. The underlying reason might be that duration of sunshine could affect the survival and transmission of the virus in the environment and it was also possible that during the days with longer duration of sunshine, children may spend less time outdoors (38), and then reduced the transmission opportunity.

Our analysis was among the few studies to examine the effects of the weather variables among different population groups (28). We found that sex, age, and occupation might be important modifiers of the effects of temperature, relative humidity, and sunshine on children HFMD, which might be due to different daily activities, behaviors, and exposures among the different populations. Particularly, we found that younger children and scattered children had higher effect of temperature. Different from the schooling children, who spent most of their daily time in the school, these subgroups usually played in their homes or the courtyard, and were exposed to more environment exposure, which might be one reason. Another possibility pointed to the difference in immunity status among different population groups; the older children might have already been afflicted with this disease and the antibodies they would have developed would protect them from being affected (39).

For the selection of the time scales, two factors should be considered. The first consideration is the biological mechanism of the association between weather factors and the disease of interest. When there is an acute effect of the weather variables on the disease occurrence, such as within a few days, we should consider doing the analysis on a daily scale; otherwise, we can choose to use weekly or monthly data. The second one is, the number of a disease at a time unit, for instance, when there are too many days without disease occurrence, it will be better to do the analysis based on weekly or monthly observations. For our analysis, previous studies have suggested that meteorological factors might have an acute effect on HFMD (28, 35), and there were sufficient daily number of HFMD cases for us to do the daily time series analysis.

A few limitations should be considered in this study. First, our study design was ecological in nature which did not allow us to explore individual-based association (40). Second, the HFMD cases depended mainly on clinical presentation, without confirming the diagnosis by microbiological or serological tests, hence resulting in potential misdiagnosis. However, this disease was considered to be an easily recognizable disease. Third, our analysis was preliminary and exploratory; we could not exclude the possibility of a spurious finding or unmeasured confounding factors that may be associated with both weather variables and HFMD occurrence.

## Conclusions

Our study suggests that temperature, relative humidity, and sunshine may be important predictors of adolescent HFMD transmission in Rizhao. Age, sex, and occupation might be important effect modifiers of the effects.
